# Integrating cataract and trachoma surgery in South Sudan: Expanding access and strengthening inclusive eye health services

**Published:** 2025-11-22

**Authors:** Angelia Sanders, Albino Nyibong, Yak Yak Bol, Jolly Kemigabo, Francis Okello

**Affiliations:** 1 Senior Associate Director: The Carter Center, Atlanta, USA.; 2 Director of Eye Services, Ministry of Health, Juba, Republic of South Sudan.; 3 National Coordinator for PC-NTDs: Ministry of Health, Juba, Republic of South Sudan.; 4 Regional Director, Eastern Africa: Cure Blindness Project, Kampala, Uganda.; 5 Country Director: South Sudan, CBM, Juba, South Sudan.

**Combining surgical services for cataract and trachomatous trichiasis is helping to extend the reach of eye health services in underserved areas.**

**Figure:**
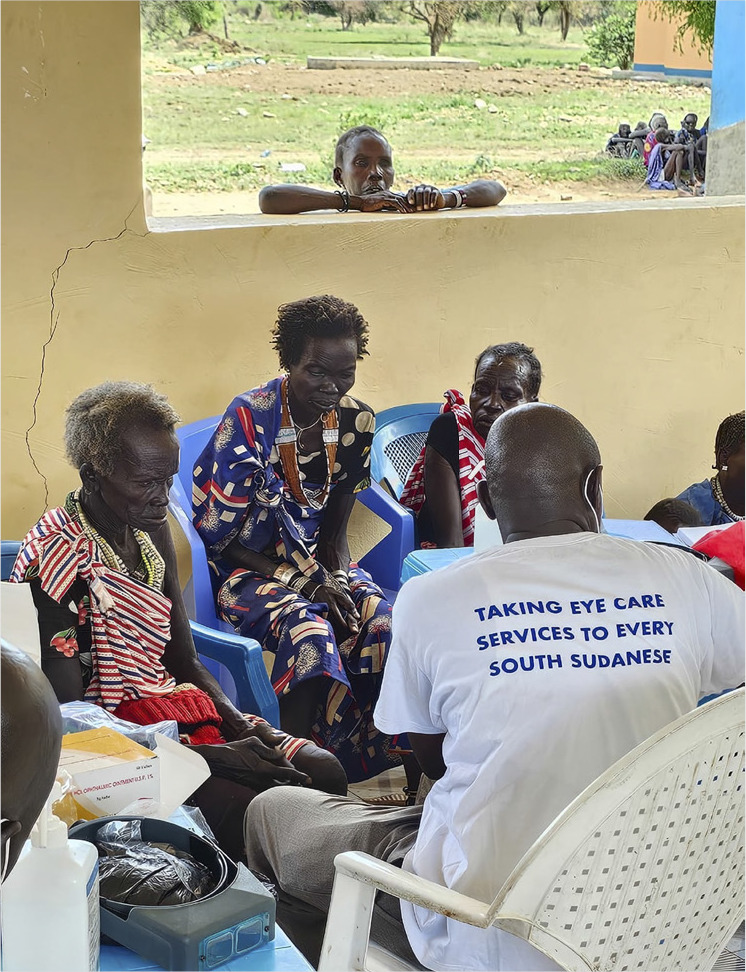
Patients being screened for cataract and trachomatous trichiasis (TT). SOUTH SUDAN

© XXXXXXXXXXXXXXXX CC BY-NC-SA 4.0

South Sudan has one of the highest burdens of preventable blindness in the world. Cataract and trachoma are both widespread, yet access to surgical services is severely limited due a shortage of trained personnel, which is exacerbated by conflict and a fragile health system. In 2024, only five ophthalmologists were practicing in the entire country, most of them in the capital, Juba. For the vast rural population, which are disproportionately affected by eye health issues, services are often out of reach.

To improve access to eye health services, South Sudan's Ministry of Health has partnered with The Carter Center, Cure Blindness Project, CBM, and the Ophthalmological Association of South Sudan to provide more comprehensive eye care services during surgical camps. These “TT+” campaigns combine surgical services for cataract and trachomatous trichiasis (TT; the blinding stage of trachoma) in one outreach effort, maximising the use of scarce resources and reaching patients who might otherwise go untreated.

The TT+ model is built on the principles of efficiency and equity. By bringing together surgical teams, supplies, and community mobilisation systems, the same outreach platform can address the country's most pressing eye health needs. This integration reduces duplication of effort by enabling the same people to screen for multiple conditions at once, lowers operational costs by transporting supplies together and deploying medical staff across multiple activities, and allows patients to receive holistic care rather than being referred back and forth between parallel programmes operating at different times. For example, a patient who has walked hours to reach a clinic for an eye care campaign is more likely to have their needs met when multiple types of surgery are available.

Community engagement is a defining strength of the approach. Chiefs, teachers, churches, health workers, Boma Health Initiative workers, political leaders, and local radio were all involved in advocacy and mobilisation. These measures build trust, increase awareness, and help ensure that patients are motivated to travel (often long distances) to reach the camps. However, delivering integrated surgery in South Sudan also highlights significant challenges that must be considered:

- **Infrastructure and logistics.** Poor road and airstrip networks, unreliable electricity, seasonal flooding, and lack of consumables and equipment complicates outreach campaigns and can make them more expensive. Transporting microscopes and surgical kits between many different remote locations increases wear and tear, and temporary facilities often lack the ventilation, lighting, and space needed for high-volume surgery.
- **Children's eye health.** An unexpected number of children are being found with TT or congenital cataract. Cultural practices and traditional treatment methods hinder early health-seeking behavior. Often, by the time children reach the hospital, it is too late to save their eyesight. These children require referral to specialised hospitals, underscoring the need for stronger pediatric eye care planning.
- **Monitoring, evaluation, and data use:** Tracking patient outcomes across multiple remote locations remains challenging, yet it is critical to collect reliable data on surgical outcomes, postoperative complications, and patient follow-up.
- **Gender equity.** Women and girls are disproportionately affected by trachoma and cataract, but often face cultural and logistical barriers to accessing surgery. Ensuring gender-sensitive outreach, transport support, and female surgical staff participation can improve equitable access.

South Sudan urgently requires investment in training eye health professionals, expanding static facilities, strengthening supply chains, and improving data systems. Greater efforts are also needed to raise awareness and mobilise existing community health structures to identify and refer children for treatment. Without these actions, the backlog of preventable blindness will continue to grow.

Integrating cataract and trachoma surgery in South Sudan represents more than a technical solution. It is a step towards universal eye health coverage in one of the most challenging environments in the world. By combining resources, engaging communities, and leveraging trachoma platforms, the TT+ model offers a practical pathway to reduce preventable blindness. The lessons from South Sudan can guide other countries seeking to extend the reach of eye health services in underserved areas.

